# Evaluation of SLS 3D-Printed Filter Structures Based on Bionic Manta Structures

**DOI:** 10.3390/ma15238454

**Published:** 2022-11-27

**Authors:** Benedikt Adelmann, Tobias Schwiddessen, Babette Götzendorfer, Ralf Hellmann

**Affiliations:** Applied Laser and Photonics Group, Faculty of Engineering, University of Applied Sciences Aschaffenburg, Wuerzburger Strasse 45, 63734 Aschaffenburg, Germany

**Keywords:** additive manufacturing, selective laser sintering, bionic structures, manta ray, filtering

## Abstract

We report on additively manufactured filter systems based on bionic manta ray structures and evaluate their filter performance. The filters are periodic lamella structures produced by selective laser sintering using PA12 polyamide powder. Two different lamella types are investigated, which are derived from two manta ray genera, namely, *Mobula tarapacana* and *Manta birostris*. The precipitator efficiency of sand particles in water is determined for both flow directions, which are referred to as the “wing” and “spoiler” arrangements. With a flat filter design, more than 90% of sand particles can be removed from the water. The variation of the lamella distance reveals that the filter effect is based on the different dynamic flow of particles and water rather than filtering by the hole size. The successful transformation of the primary flat filter design into a round filter structure is demonstrated with precipitator efficiencies above 95% and a ratio of filtered to unfiltered water of 1:1 being achieved, depending of the gap between the filter and the surrounding pipe. A shortening of the filter structure results in an unaltered precipitator efficiency but a lower ratio of filtered water. These results reveal the peculiar possibility to produce 3D round-shaped filters based on manta ray structures with additive manufacturing, achieving good precipitator efficiencies.

## 1. Introduction

Inspired by the freedom of design, which has become actionable by additive manufacturing (AM), innovative construction of lightweight [1,2,3] and lattice structures [4,5] as well as function integration have been demonstrated [6,7]. Particularly bionic-enthused designs are attractive for AM to exploit the full potential of the AM technologies [8], the approaches again often being based on lattice and lightweight structures [9,10]. Exemplifying applications, bionic inspired AM designs have been shown for automotive [11], materials engineering [12], and medical technology [13]. With respect to filter applications, the potential of AM has been utilized to realize novel filter designs and to control the permeability of porous structures [14,15,16,17]. In particular, Götzendorfer et al. [18] demonstrated fully tunable anisotropic permeability with a possible spatial flow ratio control employing laser powder bed fusion (LPBF), building specifically designed lattice structures using stainless steel.

Solid–liquid filtration is found in numerous industrial and medical applications, as well as in biological systems [19], with sieve [20,21], cross-flow [22,23,24,25], hydrosol [26], and cyclonic separation [27] being the main underlying filtration mechanisms. As the most common approach, during sieving, a liquid containing particles typically passes perpendicularly through a filter structure with regular pores or a filter medium, with the particles being retained, while the liquid is drained. Sieve filters exhibit pore sizes smaller than the particle size and thus inevitably clog in use. This, in turn, reduces filtration performance, increases resistance to filtration, and leads to downtime for filter replacement in technical applications. Other approaches may reduce but also do not completely eliminate clogging.

An intriguing filter that avoids clogging and allows for filter pores larger than the particles to be filtered is inspired by manta rays. Manta rays nourish themselves with zooplankton, microcrustaceans, and mesoplankton [20,28,29] by swimming with open mouths and separating these organisms from seawater via crossflow filtration, preventing clogging of their filtering apparatus, the gill raker [24,30], which in turn consists of parallel arrays of filter lobes [31,32,33]. During swimming, the seawater flows into the oral cavity, over the filter lobes, and filtered water without microorganisms is ejected into the sea [34]. The unfiltered water continues to flow over the filter lobes, being continuously enriched with microorganisms that are finally delivered into the alimentary tract [31]. Depending on the orientation of the filter lobes, water impinges on these in both forward (wing-like posterior filters) and reversed (spoiler-like anterior filters) directions [32,35]. In general, here, a deviation of the particle movement from the flow line of the water is considered as the solid–liquid filtration effect.

Combining physical modeling of fluid dynamics, Divi et al. [35] showed that manta rays use a unique solid–fluid separation mechanism in which direct interception of particles with wing-like structures causes particles to ricochet away from the filter pores. This filtration mechanism separates particles smaller than the pore size (which in turn can exhibit larger pore sizes), allows high flow rates (high Reynold numbers), and resists clogging. In addition, a three-dimensional (3D)-printed model of an array of filter lobes was manufactured based on anatomical parameters using color jet printing and UV curable resin, with a filtration efficiency for 1D structures being measured in the range between 19% and 62% depending on the filter lobes’ orientations. In particular, precipitator efficiencies of 19% for the “wing” and 62% for the “spoiler” were documented [35].

Li et al. [36] took advantage of this filtering approach and imitated the manta ray gill rakers using an aligned electrospun nano-fibrous silk fibroin membrane for oil–water separation. It was shown that while the water permeated through the membrane, oil was rejected by the membrane and collected in the middle pipe. Compared to traditional super-hydrophilic membrane separation conducted by the gravity-driven dead-end approach, this method can avoid fouling issues and function continuously.

Against this interdisciplinary background, this work addressed the abstraction and adaptation of the manta ray’s filter system to the additive manufacturing process. First, selective laser sintering was used to manufacture functional filter lobes in wing-like and spoiler-like orientations with varying dimensions and angles of orientation. Using an ad hoc designed experimental setup, the filter operation was evaluated and quantified in terms of filtering efficiency to identify optimized geometries. Moreover, the design of the filter structure was generalized to a 3D round filter structure integrated into a pipe, demonstrating the full engineering implementation of the concept.

## 2. Experimental

### 2.1. SLS 3D Printing

Unlike conventional filters, the specific filter mechanism of the manta ray allows for the filtering of particles smaller in size than the pore size, and thus selective laser sintering (SLS) was chosen for this study. Though contrivable pore sizes for stable, large scale filter structures are limited to about 800 μm, with the employed dynamic cross filtering mechanism, even significantly smaller particles can be separated.

The system used for SLS was a Formiga P110 with a building chamber of 200 × 250 × 330 mm^3^; in size (EOS GmbH, Krailing, Germany), equipped with a 30 W CO_2_ laser (spot size of the galvo scanner at focal position is 0.25 mm). The system allows for a build rate of up to 20 mm height/hour with the layer thickness being variable between 0.06 mm and 0.12 mm. To prevent oxidation processes, the build chamber is flooded with nitrogen as a protective gas. A built-in 4-channel infrared heater (IR), monitored by a pyrometer, ensures a controlled temperature in the process chamber. The temperature in the process chamber is just below the melting point of the material used. Thus, thermal distortion can be reduced during manufacturing.

**Figure 1:**
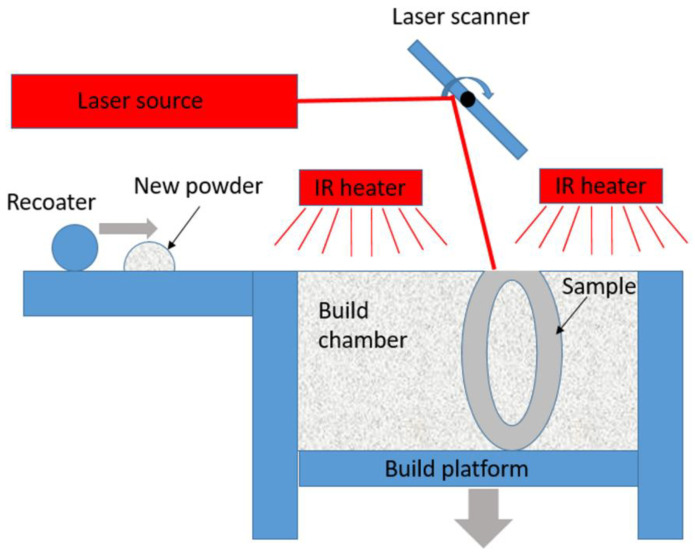
Scheme of the selective laser sintering machine.

Figure 1 schematically shows the functional setup of an SLS manufacturing unit. A coater feeds polyamide powder in layers from the powder supply container to the build platform. The layer thickness is 100 µm. A laser is positioned by a laser scanner and melts the powder selectively to form a two-dimensional component. By lowering the build platform layer by layer, a three-dimensional element is created by joining several layers.

The material used is polyamide 2200 (PA 12) [37], a thermoplastic material characterized by high strength, high dimensional stability under heat, and high chemical and wear resistance [37,38]. The powder is characterized by a size distribution between 20 μm and 100 μm.

### 2.2. Particle Properties and Measurement

**Figure 2:**
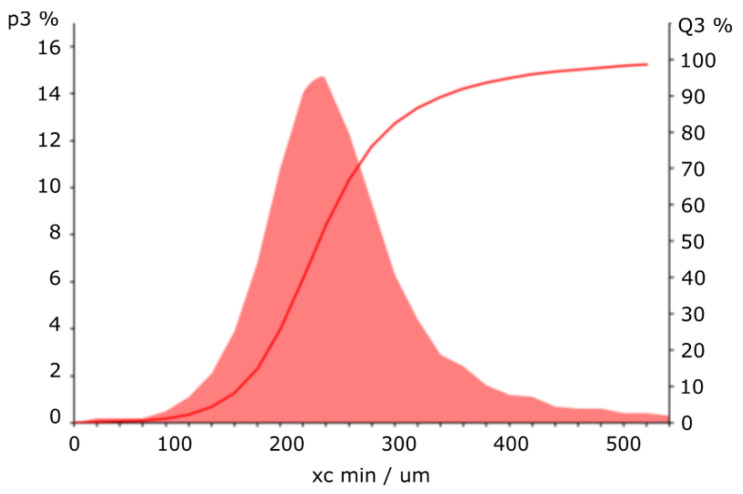
Particle size distribution of the sand added to the water (particle mass ratio Q3).

To evaluate the solid–liquid filter characteristics of the 3D-printed manta ray wing and spoiler structures, we used sand added to water (20 g, 100 g). The particle size distribution of such aqueous sand solutions is depicted in Figure 2, and was analyzed by a CAMSIZER X2 based on dynamic digital image analysis methods according to ISO 13322-2 (Retsch Technology GmbH, Haan, Germany). The quantiles of the particle distribution were determined to 166 μm (10% quantile), 233 μm (50% quantile), and 343 μm (90% quantile), respectively.

To measure the filter efficiency of the 3D-printed device, both the filtered and unfiltered water were collected, and the sand contained in them was dried and weighed. The precipitator efficiency *pe* was calculated (Formula (1)) by the ratio of the amount of sand in the unfiltered water $m_{u}$ to the complete inserted sand $m_{c}$. The sand in the filtered water was used to control the measurement to ensure that no sand remained in the experimental apparatus.

(1) $$pe=\frac{m_{u}}{m_{c}}$$

### 2.3. Filter Types and Function

**Figure 3:**
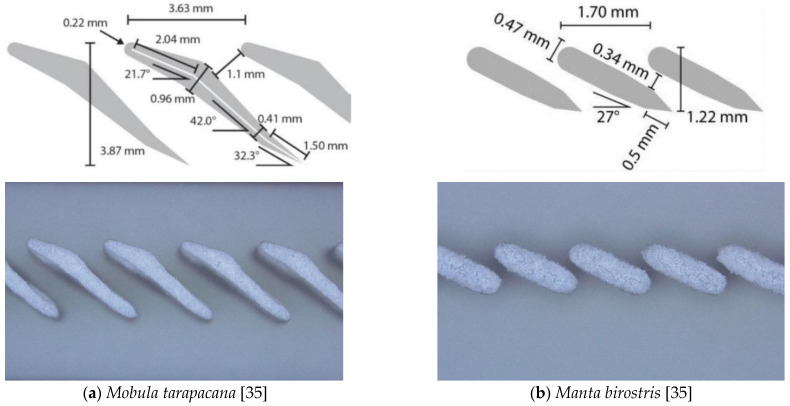
Different lamella types in technical drawings (**top**) and photos of 3D-printed structures (**bottom**).

The two different designs of the filter structures under study were inspired by the manta ray, namely, by the ray genera *Mobula tarapacana* (short: Mobula) and *Manta birostris* (short: Manta), which have different filter shapes for feeding [35]. Their basic design was transferred into a CAD model (SolidWorks) and 3D printed by SLS. Figure 3 compares the lamella designs to images taken from 3D-printed structures, highlighting that the printed structures matched well with the CAD models. Deviations in the form of curves at the lower tips were visible, which could be attributed to the minimum producible structures due to the laser beam diameter, the heat-affected zone, and the powder size.

The filter function principle has been described by Divi et al. [35], showing that the solid–liquid filtration effect of the manta ray filter lobes is based on a deviation of the particle movement from the flow line of the water. The particles rebound on the top of the lamella structure and move upwards into the water flow while water passes the gaps between the lamellae. 

In terms of particle size, the filtration effect of the structure increases significantly when the particles exceed 200 μm in diameter. The density of the particles, on the other hand, has only a minor effect on the filtration efficiency [35]. This is consistent with the bouncing effects of the particles depending on the contact forces, which do not depend on particle density.

Such filter structures only work for high Reynolds numbers larger than 900 and thus flow velocities above 0.3 m/s [32,35]. Therefore, in our experiments, a flow velocity of 0.89 m/s was chosen in order to achieve a distinct filtration effect. To validate the achieved results, five filtration experiments with the same particle quantities were carried out for each structure and arrangement.

### 2.4. Filter Setup

**Figure 4:**
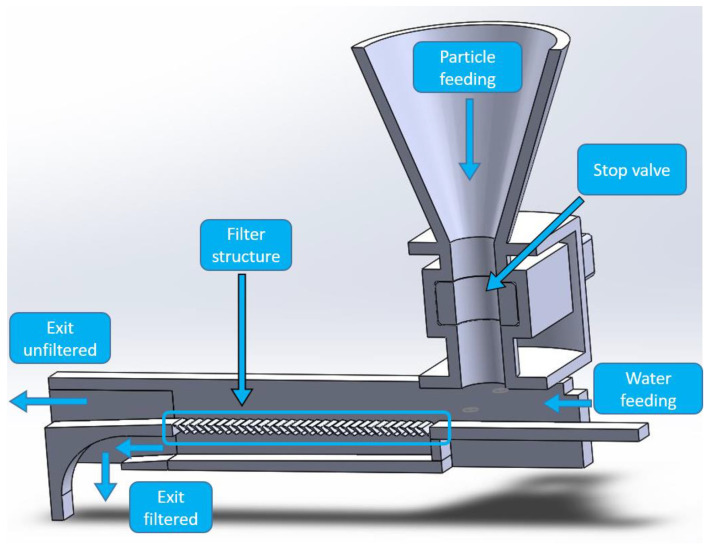
Experimental setup for measuring flat filter structures.

To evaluate the filter structures, two different measurement setups were used. The first setup, shown in Figure 4 is suitable for flat filter structures and consists of a feed line for the water and a funnel with a gate valve for targeted dosing of the sand into the water. The liquid mixed with sand passes through the horizontal filter structure, which can be rotated depending on the direction of the flow type or switched to another filter type. The unfiltered water flows over the filter structure to the outlet, where the water is collected. The filtered water flows through the filter structure and leaves the setup through the lower outlet.

**Figure 5:**
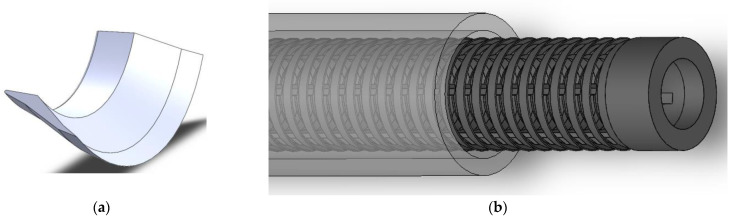
Transformation of the filter structure into a round shape. (**a**) individual printed segment; (**b**) stacked arrangement of filter elements into a pipe.

Though such 1D filter structures are suitable for fundamental studies of the filtration effect and efficiency, as generally has also been used by Divi et al. [35], to fully exploit the potential of additive manufacturing and to meet requirements of industrial implementation, we extended the manta ray filter lobe design to a fully cylindrical geometry (Figure 5). This could be inserted to hoses and pipes transporting liquids having round cross-sections with the filter area being extended as compared to a simple linear arrangement (Figure 3a). The individual, fully 3D-printed segments were stacked with defined spacing and stabilized with vertical struts. The resulting cylindrical structure filtered from the inside to the outside (Figure 5b). The filter was placed in a PMMA tube with an inner diameter of 24 mm, while the outer diameters of the filter structures were varied to change the flow dynamic. At the exit of the tube, the liquids from the inner and outer parts of the tube were collected and evaluated separately.

**Figure 6:**
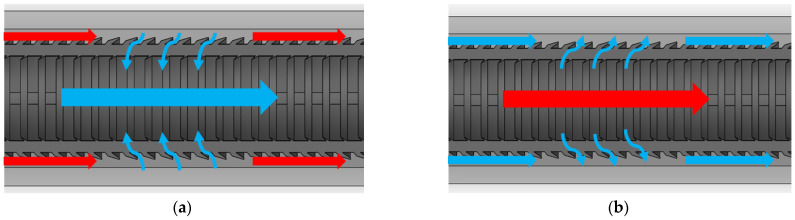
Flow of the filtered (blue) and unfiltered (red) water in the filter structure in (**a**) normal and (**b**) inverted operation.

The flow path in the cylindrical filter is shown in Figure 6. In normal operation (Figure 6a), the unfiltered water flows in the outer area of the filter and also leaves the filter in the outer area. Thus, the filter operates in the spoiler mode. The filtered water flows through the filter into the inner area and leaves the structure through the inner exit. The filter can also be used in the inverted mode (Figure 6b), where the unfiltered water is supplied in the inner area, and the filtered water leaves via the outer exit.

## 3. Results

### 3.1. Comparison of Filter Types and Flow Direction

Both one dimensional lamella structures (Figure 3) were tested in terms of precipitator efficiency in spoiler and wing modes. The results of the measurements are summarized in Table 1. The structure manta spoiler was characterized by good precipitator efficiency. However, a low flow rate of filtered water was measured. Similar results were achieved for the manta wing design. At first glance, this geometry had an excellent filtration effect at medium flow rates. However, due to the gap orientation in the direction of flow, particles became caught between the lamella structures and clogged the gaps. This resulted in a loss of filtration performance, and the filtration process was not reproducible due to the remaining sand in the filter. The orientation of the filter lobes in the direction of flow in the mobula wing design ensured that the water only partially overflowed the filter surface. The main part of the water and particles passed through the gaps of the filter and ended up in the filtered water tank. This led to an absence of any filtration effect. Finally, the mobula spoiler structure showed the best filtering results. It had a good filtration effect and ensured a sufficient flow of filtered water. Additionally, this filter structure was tested with 100 g sand in the water, and the precipitator efficiency stayed at 90.1 ± 1.3%, which was almost as high as the 20 g sand experiments. Since several test series revealed no difference in the filtration rate between these added quantities of sand, the results of 20 g sand per experiment were used for all further examinations. This confirmed that the structure achieved its filtration effect even with higher particle quantities, and no clogging effect was observed. On the basis of these results, this structure was selected for further consideration.

### 3.2. Variation of Lamella Spacing 

**Figure 7:**
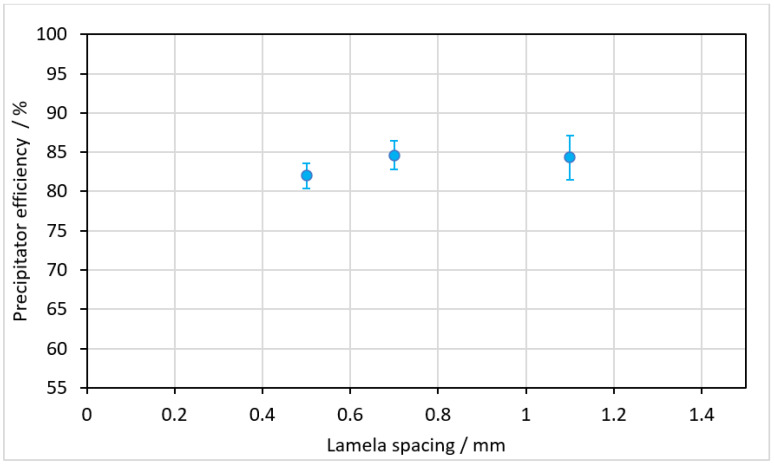
Precipitator efficiency as a function of smaller lamella distance.

According to the simulations of Ref. [35], a reduction of the lamella spacing with otherwise unchanged flow conditions will not result in an improvement of the filtration effect. We therefore altered the lamella spacing of the mobula spoiler geometry between 1.1 mm and 0.5 mm, resulting in a reduction of the gap size. Please note that a lamella spacing of 1.1 mm was used in the previous measurements. The measured precipitator efficiency for different lamella spacing is depicted in Figure 7. The results revealed that by reducing the lamella spacing, no improvement in the filtration effect was achieved as compared to the initial arrangement. This is in accordance with the results of Divi et al. [35], i.e., the filter effect was based on the dynamic flow of particles and water and not like a mechanical filter at the gap of the lamellae. Please note that the difference between the precipitator efficiency in Figure 7 and in Table 1 is attributed to a chanced cleaning procedure between subsequent experimental tests in both test series.

### 3.3. Flat Filter Structure Rotation Angle

**Figure 8:**
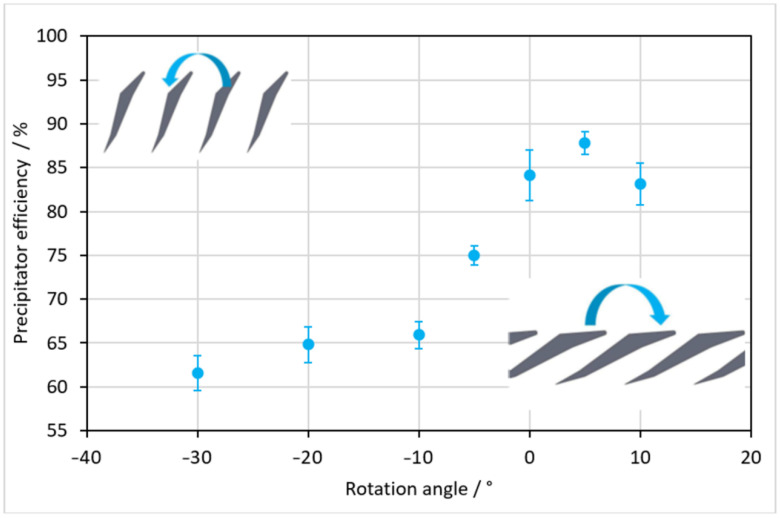
Precipitator efficiency of the flat filter depending on the lamella rotation angle.

In order to optimize the filter, the influence of the angle of orientation of the lamellae was investigated. Due to the high flexibility of the 3D printing process, rotated lamellae can easily be produced. As described before, the particles contained in the water bounce off the trailing edge of the lamellae. A change in the position of this edge could therefore influence the trajectory of the particles. To investigate this, the lamella angle of the mobula spoiler structure was varied in the range between 10° and −30° with respect to the standard orientation shown in Figure 3b, which represents the angle of 0°. To illustrate the direction of rotation, two exemplifying illustrations are included in Figure 8.

A maximum rotation of +10° in the positive direction was chosen, since at larger angles, the rebound edge would run horizontally to the flow and would therefore no longer show any influence. A minimum angle of −30° in the negative rotation direction was chosen because with higher rotation, the rebound edge would be perpendicular to the flow direction. The filtration effect as a function of the lamella rotation is shown in Figure 8. A continuous increase in the filtration effect was observed between rotation angles of −30° and +5°. This means that a steeper orientation of the structure to the flow would lead to a deterioration of the filtration effect. At an angle of +5° the precipitator efficiency would peak, being higher compared to the standard orientation. As a consequence, with a rotation angle of +5°, the filter would be improved slightly, so this angle was used in the subsequent experiments. With a further increase of the angle, the filtration effect dropped again, which confirmed the theory of the rebound trajectories of the particle being too flat, which reduced the filtration effect.

### 3.4. Round Filter Structure Depending on the Filter Size

Similar to the afore-presented results, for the fully cylindrical filter geometry (Figure 5) inserted to a tube, the precipitator efficiency was also determined. In addition, the ratio between filtered and unfiltered water was measured. To optimize the round filter structure, the outer diameter of the cylindrical filter was varied at 17 mm, 19 mm, and 21 mm. At the same time, the inner diameter of the surrounding PMMA tube was kept constant at 24 mm. This resulted in a reduction of the gap between the outer diameter of the filter and the inner diameter of the tube as the filter diameter increased (see Figure 5). In a further examination, the filter structure was used in the inverted operation mode according to Figure 6b.

**Figure 9:**
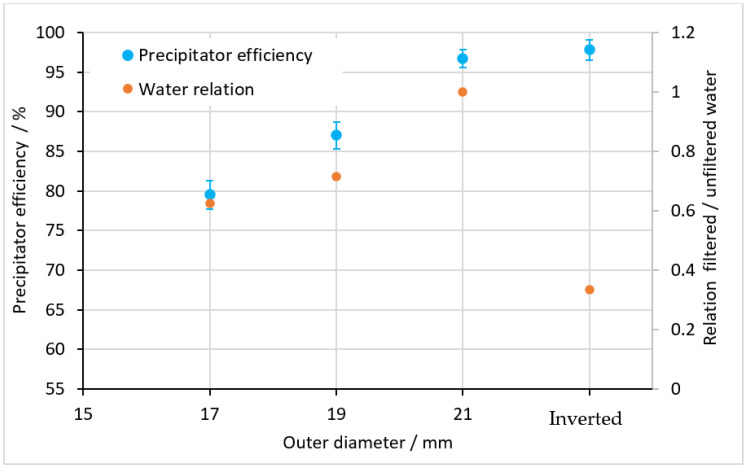
Precipitator efficiency and ratio of filtered to unfiltered water for the round filter structure depending on the outer diameter of the filter.

The results shown in Figure 9 reveal improved precipitator efficiency with increasing structure diameter, i.e., a decreasing gap between the structure and the inside of the tube. With regard to the filtration effect, the inverted structure was at a similar level compared to the structure diameter of 21 mm and higher compared to the flat filter.

With regard to practical applications of the filter tube geometry, additional to the filtration effect, the flow rate of filtered and unfiltered water must be considered. The results shown in Figure 9 reveal an improving flow ratio of filtered water with increasing filter diameter. In particular, while the precipitator efficiency of the inverted filter structure was similar to that of the 21 mm structure, its flow ratio was significantly worse. As a result, the adaptation of the initially flat manta ray structure into a circumferential tube structure can be described as successful, with good precipitator efficiency and a good filtered water ratio. In comparison with other manta ray-based filters, our precipitator efficiency, which was above 95%, was quite high compared to the measured literature values of 19% for “wing” and 62% for “spoiler” arrangements [35]. Please note that the results are difficult to compare due to the different experimental setups.

### 3.5. Filter Length Dependence of the Round Filter Structure

Because the available space for a filter in technical applications is an important decision criterion, the influence of the filter length must be determined. Therefore, a filter diameter of 21 mm was chosen because of the best filtration effect and the best flow ratio, as indicated in the previous section.

**Figure 10:**
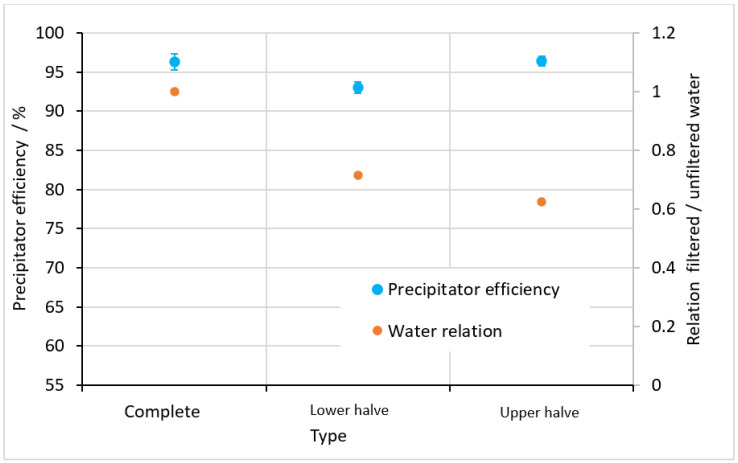
Precipitator efficiency and ratio of filtered to unfiltered water for the round filter depending on the filter length.

To determine the influence of the filter length, the original filter length of 87 mm was halved to 43.5 mm. In the test setup, the upper or the lower halves of the original filter length were designed as the filter area, while the other half was closed off. The results shown in Figure 10 revealed that a reduction of the filter length led to no significant variation of the precipitator efficiency. Simultaneously, the ratio of unfiltered to filtered water increased, which is disadvantageous. As a consequence, in order to build a small filter, the length can be reduced with unaltered precipitator efficiency but there would be a disadvantageous ratio between unfiltered and filtered water.

### 3.6. Particle Size Distribution after the Filter

To characterize the filter quality, the remaining sand’s particle size distribution in the filtered water was analyzed in the same way as in Figure 2. The results of the quantiles in the size distribution are shown in Table 2. The Q 50% quantile is the mean of the size distribution, and the Q 90% and Q 10% give the upper and lower 10% of the distribution, respectively. The measurements revealed that for filters with low precipitator efficiency such as the flat filter with −30° rotation (cf. Figure 8), the Q 10% and Q 50% quantiles remained almost constant, while the Q 90% quantile was reduced remarkably. This means that the filter mainly filtered the largest sand particles significantly more than the small particles. Comparing the round filters, it is obvious that for filters with high precipitator efficiency (cf. Figure 9), all quantiles were reduced, so the sand became finer with increasing precipitator efficiencies.

## 4. Conclusions

The results of this publication reveal the possibility of producing 3D round-shaped filter systems based on manta ray structures with selective laser sintering that are installable into hoses and pipe systems. The filters are periodic lamella structures adapted from the manta ray genera *Mobula tarapacana* and *Manta birostris*. The evaluation of filtering sand from water reveals advantages of the *Mobula tarapacana* structure in the spoiler configuration due to a higher precipitator efficiency and a higher flow rate of filtered water. The insignificant dependence of the precipitator efficiency from the lamella spacing confirms that the main filter effect is based on the different dynamic flows of particles and water rather than on the minimal gap size. The lamella structure is transformed into a pipe geometry and reveals precipitator efficiencies above 95% at a filter diameter of 21 mm and a ratio of filtered to unfiltered water of 1:1. In conclusion, additive manufactured filters adapted from manta rays can be produced in pipe-shaped geometries achieving high precipitator efficiencies.

## Figures and Tables

**Table 1:** Precipitator efficiency and filtrate flow depending on the filter type and flow direction.

Name	Scheme 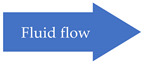	Precipitator Efficiency	Filtered Water Flow
Manta Spoiler	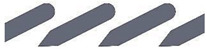	93.2 ± 0.98	Low
Manta Wing	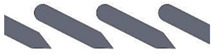	99.3 ± 0.47	Medium
Mobula Spoiler	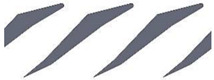	91.5 ± 0.84	High
Mobula Wing	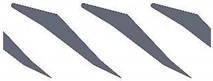	0	High

**Table 2:** Particle size distribution of filtered water after using different filters.

Filter Type	Q 10%/µm	Q 50%/µm	Q 90%/µm
Before filter	166	233	343
Flat filter mobula “spoiler”	152	215	284
Flat filter −30° rotation	163	226	304
Round filter 17 mm	161	224	303
Round filter 19 mm	150	214	283
Round filter 21 mm	137	200	263
Round filter inverted	143	204	266
